# Supplementary material for: Modeling glioblastoma heterogeneity as a dynamic network of cell states
Source: Mol Syst Biol. 2021 Sep 16;17(9):e10105. doi: 10.15252/msb.202010105 (PMC8444284; doi:10.15252/msb.202010105)
Supplement: Supplementary file 6 — Source Data for Figure 5 [file MSB-17-e10105-s004.zip › Figure5A_sourcedata/GSEA_3017/hallmarks_stateC.GseaPreranked.1621934520804/HALLMARK_ESTROGEN_RESPONSE_EARLY.html]

Details for gene set HALLMARK\_ESTROGEN\_RESPONSE\_EARLY[GSEA]

|  || Dataset | state13017 |
| Phenotype | NoPhenotypeAvailable |
| Upregulated in class | na\_neg |
| GeneSet | HALLMARK\_ESTROGEN\_RESPONSE\_EARLY |
| Enrichment Score (ES) | -0.29740614 |
| Normalized Enrichment Score (NES) | -1.332273 |
| Nominal p-value | 0.14886731 |
| FDR q-value | 0.16838504 |
| FWER p-Value | 0.843 |
Table: GSEA Results Summary

  

Fig 1: Enrichment plot: HALLMARK\_ESTROGEN\_RESPONSE\_EARLY      
 Profile of the Running ES Score & Positions of GeneSet Members on the Rank Ordered List

  

| PROBE | GENE SYMBOL | GENE\_TITLE | RANK IN GENE LIST | RANK METRIC SCORE | RUNNING ES | CORE ENRICHMENT || 1 | RHOBTB3 |  |  | 124 | 0.313 | -0.1787 | No |
| 2 | RBBP8 |  |  | 177 | 0.267 | -0.2374 | No |
| 3 | KAZN |  |  | 200 | -0.252 | -0.2457 | No |
| 4 | SEC14L2 |  |  | 202 | -0.252 | -0.2173 | No |
| 5 | FDFT1 |  |  | 243 | -0.262 | -0.2557 | No |
| 6 | MYOF |  |  | 251 | -0.263 | -0.2364 | No |
| 7 | UGCG |  |  | 287 | -0.273 | -0.2649 | Yes |
| 8 | ABHD2 |  |  | 296 | -0.275 | -0.2460 | Yes |
| 9 | DHCR7 |  |  | 322 | -0.285 | -0.2556 | Yes |
| 10 | DLC1 |  |  | 347 | -0.296 | -0.2620 | Yes |
| 11 | ABLIM1 |  |  | 348 | -0.297 | -0.2266 | Yes |
| 12 | FOS |  |  | 377 | -0.309 | -0.2385 | Yes |
| 13 | SLC2A1 |  |  | 383 | -0.311 | -0.2101 | Yes |
| 14 | MYC |  |  | 421 | -0.336 | -0.2345 | Yes |
| 15 | RAB31 |  |  | 445 | -0.349 | -0.2330 | Yes |
| 16 | HES1 |  |  | 470 | -0.373 | -0.2302 | Yes |
| 17 | RETREG1 |  |  | 473 | -0.375 | -0.1889 | Yes |
| 18 | ENDOD1 |  |  | 493 | -0.405 | -0.1738 | Yes |
| 19 | PRSS23 |  |  | 510 | -0.438 | -0.1494 | Yes |
| 20 | GJA1 |  |  | 552 | -0.527 | -0.1579 | Yes |
| 21 | PODXL |  |  | 559 | -0.546 | -0.1032 | Yes |
| 22 | CD44 |  |  | 575 | -0.659 | -0.0507 | Yes |
| 23 | CA12 |  |  | 586 | -0.717 | 0.0174 | Yes |
Table: GSEA details [plain text format]

  

Fig 2: HALLMARK\_ESTROGEN\_RESPONSE\_EARLY: Random ES distribution      
 Gene set null distribution of ES for **HALLMARK\_ESTROGEN\_RESPONSE\_EARLY**
